# Kelvin Probe Microscopy Investigation of Poly-Octylthiophene Aggregates

**DOI:** 10.3390/ma15031212

**Published:** 2022-02-06

**Authors:** Joaquin Bermejo, Jaime Colchero, Elisa Palacios-Lidon

**Affiliations:** Centro de Investigación en Óptica y Nanofísica (CIOyN), Departamento de Física, Universidad de Murcia, 30100 Murcia, Spain; joaquin.bermejo@phys.ens.fr (J.B.); colchero@um.es (J.C.)

**Keywords:** Kelvin probe microscopy, surface potential, semiconducting polymers, poly-3-alkylthiophene

## Abstract

Conductive polymers have fundamental relevance as well as novel technological applications in the organic optoelectronics field. Their photophysical and transport properties strongly depend on the molecular arrangement, and nanoscale characterization is needed to fully understand the optoelectronic processes taking place in organic devices. In this work, we study the electrostatic properties of poly-3-octylthiophene isolated structures: disordered low-packed polymer chains and crystalline layered lamellar assemblies. We characterize the electronic ground state using Kelvin probe microscopy. This allows us to resolve a rich variety of surface potential regions that emerge over the different polymer structures. These *SP* regions are correlated with different molecular aggregates.

## 1. Introduction

**Figure 1:**
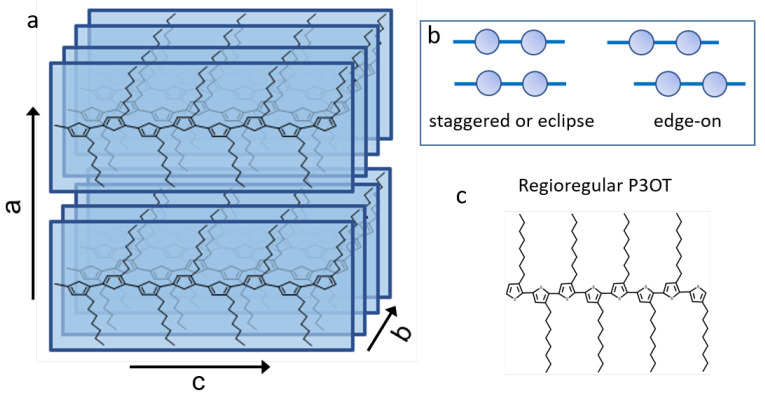
(**a**) Schematic structure of a crystalline P3OT aggregate. (**b**) Two possible PAT aggregates depending on the relative polymer chain shift along the c axis. (**c**) Representation of a P3OT chain.

Semiconducting conjugated polymers (SCPs) are widely used in electronic devices such as solar cells, organic field effect transistors and light-emitting devices [1,2,3]. Their potential lies in their tunable optoelectronic properties, low cost and easy production methods, which have boosted the development of flexible opto-electronic platforms [4]. The final application of SCPs strongly relies on the charge transport characteristics, the carrier mobility as well as on the photophysical properties. In polymer aggregates, the system is either 2D or 3D and all these properties are mediated by the competition between interchain (along the polymer chain) and intrachain interactions (between polymer chains). These interactions are modeled taking into account two main contributions: on the one hand, the coulombic dipole–dipole coupling between adjacent polymer units (along the same chain or between chains) [5], and, on the other hand, the charge transfer (CT) interaction, which depends on the wave function overlapping between adjacent polymer chains [6]. Depending on the positive (negative) sign of these interactions, the SCP aggregates have been traditionally classified as H-aggregates (J-aggregates). This leads to a rich variety of aggregate types, named HJ, JH, HH, JJ, in which the first label is related to the dipolar coupling sign and the second to the sign of the CT interaction [6,7,8,9]. Traditionally, SCP aggregates have been discriminated by analyzing the characteristic features of absorption and emission spectra. However, very little information about the relative strength of these interactions is gained from optical measurements [10], which, in addition, determine the electronic structure of the ground state changing the HOMO and LUMO energies and therefore the charge transport properties [6,7]. In fact, polymer aggregates with similar optical spectra but different work functions or aggregates with different optical signatures but almost similar work functions may be found [11,12,13]. Therefore, the characterization of the SCP aggregates’ ground state is key to gain control over the relationship between structure, morphology and electronic properties for the design of SCP assemblies. Among the SCPs, the poly-3-alkylthiophenes (P3ATs) and, in particular, poly-3-hexylthiophene (P3HT) have attracted particular attention due to their good solubility in organic solvents and strong tendency to self-assemble in π-stacked quasi-crystalline lamellae [14]. This confers upon them interesting photophysical and charge transport properties, with many potential applications in optoelectronic devices [14,15]. The schematic P3AT crystalline structure is shown in Figure 1. Adopting the common convention, the a-axis is the direction of the alkyl-side groups, the b-axis is the π−π stacking direction, and the c-axis corresponds to the polymer chain backbone [16,17,18,19,20]. In general, the thermodynamically favored orientation of the crystalline structure on a substrate consists of polymer chains stacked with the alkyl side chains lying perpendicular to the substrate. It is known that efficient charge transport occurs in the c-b plane, along either the backbone direction (c-axis) or the π−π stacking direction (b-axis), whereas alkyl side chains act as charge barriers along the a-axis [20,21].

This picture of the crystalline lamellar P3AT structures is too simple to explain the different characteristics found in different samples. As stated above, the electronic structure of the ground state and the HOMO level position is determined by the competition of intrachain–interchain interactions, and a small variation in the molecular packing may drastically modify it [6,7]. While intrachain effects are related to the conjugation length and depend on the planarity of the semi-rigid polymer backbone chain, interchain interactions depend on the registration distance along the b-axis, as well as on the relative chain slip along the c-axis: two extreme situations occur when the thiophene rings of adjacent polymer chains are facing each other (in a staggered or eclipse aggregation), or when the chains are displaced by half a tiophene ring length (edge-on aggregation) (Figure 1b). While the first configuration enhances π−π overlapping and thus CT interaction, the latter hinders it [6].

As outlined in the review by Tremel and Ludwigs [20,22,23], it has been largely investigated how molecular weight and chain regularity [24,25,26], as well as the processing method and solvent [18,27,28,29,30,31], influence the chain order and how these parameters affect the transport properties [24,27,28,30,32,33]. In addition, a model to distinguish between H-aggregates and J-aggregates from spectroscopic measurements has been proposed [34,35].

It is well known that the solvent evaporation effects during sample preparation critically condition the polymer chain kinetics [20,25]; correspondingly, it is not surprising that the final polymer aggregation strongly depends on the preparation method, as well as on the solvent and on the substrate used for deposition. To overcome this problem, the use of P3HT nanostructures such as nanowires [36,37] and nanoparticles [38,39,40] with a pre-established aggregate structure in solution is gaining attention. They have the advantage that their internal aggregation structure remains unchanged when processed into films, increasing sample reproducibility.

The characterization of P3AT thin films is usually addressed with macro- or microscopic techniques such as conductivity [24,27,28,30,32,33], X-ray diffraction [16,19,41], absorption and photoluminescence optical techniques [12,37,42], etc. These techniques average over a large sample region, blurring the material inhomogeneities that may take place at the nanomectric scale. In this case, the electronic properties will be determined by the properties of each individual region, as well as the phenomena that take place at the grain boundaries between different regions. Scanning force microscopy (SFM) techniques, and in particular Kelvin probe microscopy (KPFM), are ideal non-invasive tools to characterize them. They allow us to discern the nanoscale substructure of semiconducting polymer thin films [25,43,44] and to characterize their electronic ground state through surface potential (*SP*) measurements that can be directly correlated with the local work function [12,45,46,47].

In this work, we address the nanoscale morphological and electrostatic properties of poly-3-octylthiophene-2,5-diyl (P3OT) crystalline aggregates by means of KPFM. To obtain isolated crystalline regions, as well as to have the substrate as a reference, we have used sub-monolayer low-covered samples. Instead of the widely studied P3HT, we have chosen P3OT due to its higher tendency to form crystalline regions, which offers a suitable system to study the fundamental properties of SCPs. We focus on the ground state characterization, correlating the surface potential (*SP*) with the polymer chain aggregation within structures that self-assemble during the solvent de-wetting. To study the substrate dependence of the polymer aggregation process, we have used two very different substrates: highly oriented pyrolytic graphite (HOPG) and flat indium tin oxide (ITO). From a morphological perspective, we have found two well-differentiated types of polymer structures: isolated lamellar structures that are composed of one or several layers with a well-defined high and a thin loosely packed disordered chain film that partially covers the substrate. The electrostatic characterization shows a diverse variety of surface potential regions, not directly correlated with topography features that can be ascribed to different chains’ packing. Surprisingly, we have obtained very similar results for both HOPG and ITO substrates, indicating that most of the findings are intrinsic polymer properties rather than substrate-induced effects. Therefore, we only show results obtained on HOPG, while those for ITO are included as Supplementary Material.

## 2. Materials and Methods

### 2.1. Sample Preparation

**Figure 2:**
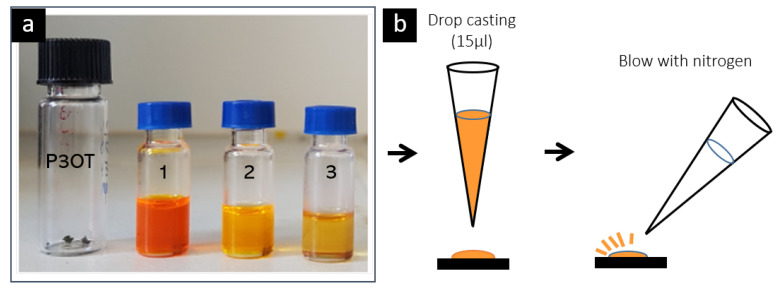
(**a**) P3OT dispersions on toluene. Dispersion 3 is used for sample deposition. (**b**) Scheme of the sample preparation.

The P3OT samples were prepared from toluene dispersions. Regioregular P3OT (SigmaAldrich Mn = 34,000) was used as received, without any further treatment. These P3OT polymers have an average chain length of around 64 nm, a height of 2.1 nm (in the direction of the alkyl side chains) and a thickness of 0.7 nm. To prepare dissolutions, 16 μg of P3OT was dispersed in 1 mL of toluene. Then, this solution was further diluted two times until a concentration of $1.6\times 10^{-2}$ mg/mL was reached. The bright orange color of the dispersion indicated the absence of polymer aggregates in the solvent as aggregate formation is accompanied by a color change to purple/violet (see Figure 2). A 15 μL drop of this final dispersion was cast on the substrate. To obtain low surface coverage, the sample was blown with nitrogen before the toluene was completely dried (Figure 2). We used two different substrates: highly oriented pyrolytic graphite (HOPG), which was previously exfoliated with scotch tape in order to have a fresh clean surface, and ultraflat indium tin oxide (ITO), which was previously cleaned with ethanol before deposition.

When working with a diluted dispersion and a low-coverage surface, it is fundamental to verify that the structures found on the sample are related to polymer features and not to the solvent impurities. Thus, we first characterized the substrate surface after casting a drop of pure toluene (see Figure S1 in the Supplementary Material).

### 2.2. Sample Characterization and Data Processing

Experiments were performed at room temperature and ambient conditions with a commercial SFM from *Nanotec Electrónica*. *WSxM* software was used for data acquisition and processing [48]. Platinum tips (Olympus, k = 2 N/m, $f_{o}\approx$ 75 kHz) were used. Imaging was carried out in non-contact amplitude modulation dynamic mode (AM-DSFM) with the Phase Locked Loop (PLL) board of our electronics enabled. The tip–sample system was then always at resonance and the frequency shift (Δf) from the free resonance frequency—induced by force gradients of tip–sample interaction—can be measured as an additional channel of (chemical) information. This mode is considered a variant of the traditional AM-DSFM mode, where the phase signal is allowed to vary [49]. We used a small oscillation amplitude ($a_{free}=5$ nm) and the set-point oscillation amplitude ($a_{set}$) was set to an amplitude reduction factor $r=a_{set}/a_{free}\approx 0.85$, in order to keep the tip–sample system in a low-interaction, attractive regime. To obtain the surface potential (*SP*), the KPFM was operated in frequency modulation mode (FM-KPFM), i.e., force gradients induced by electrostatic interaction were measured. Further details on the KPFM set-up are reported in [46].

In this work, we focused our analysis on the main topography channel, as well as on local surface potential (KPFM data) and Δf secondary channels, all recorded simultaneously. While the KPFM signal allowed us to obtain the local *SP* of the sample, the Δf signal is sensitive to chemically different materials. As explained in [46,49], the Δf signal includes conservative interactions such as Van der Waals and, under the working conditions chosen, also capillary forces due to the liquid necks being formed and ruptured during cantilever oscillation. Therefore, this signal is suitable to detect materials with different hydrophilicity. The electrostatic interaction was mainly cancelled out by the KPFM feedback and recorded (indirectly) as KPFM data.

Data analysis was carried out using the open-source Materials Morphology Python m2py library [50]. This versatile and widely adaptable code combines computer vision and machine learning techniques to recognize different sample regions as well as different material properties depending on the input channel used for the segmentation process, as explained in detail in [51]. Once the image is segmented in different regions, the properties of each region obtained from other acquisition channels can be analyzed independently.

## 3. Results

**Figure 3:**
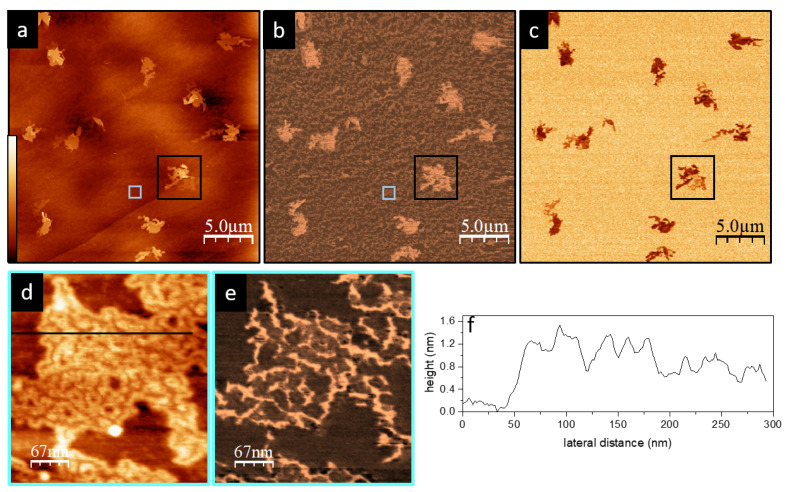
(**a**) Topography (z scale 40 nm). (**b**) Δf (z scale 180 Hz). (**c**) KPM (z scale 1 V) low-magnification images of a HOPG surface partially covered with P3OT aggregates. An example of a random and a lamellar structure, discussed in the main text, are squared in blue and black, respectively. High-magnification images (blue squared in (**a**)). (**d**) Topography (z scale 2 nm). (**e**) Δf (z scale 56 Hz) of the disordered inter-lamellar random structure. (**f**) Topography profile marked in (**f**).

A representative sample region is shown in Figure 3. The topography image (Figure 3a) confirms the low polymer coverage as the HOPG steps are still visible. Two different polymer-type structures are distinguished: on the one hand, isolated layered lamellar structures, and on the other hand, a much thinner structure that partially covers the HOPG surface. These two types of regions are better identified in the frequency shift images (Δf) (Figure 3b).

High-magnification images of the thin inter-lamellar structure (Figure 3d,e), show a wire-like disordered ensemble with a height of around 1.2 ± 0.3 nm (Figure 3f). Taking into account the dimensions of the P3OT polymer chains, these thin structures should correspond to polymer chains oriented with the tiophene rings facing the HOPG substrate and the alkyl side chains parallel to it—that is, to molecules adsorbing “flat” onto the substrate. Moreover, from the topography roughness and the appearance of the frequency shift Δf image, it is reasonable to identify this substructure with random loosely packed chains. We note that, in the KPFM image (Figure 3c), this thin random structure shows no contrast with respect to the HOPG.

**Figure 4:**
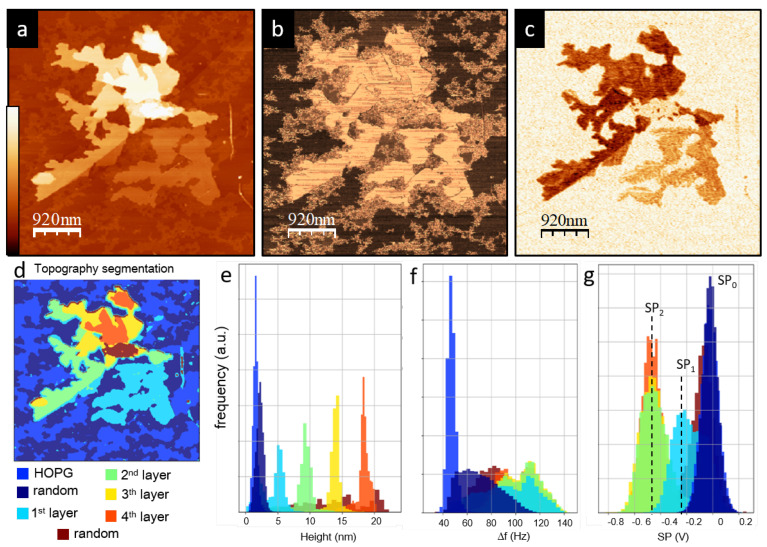
(**a**) Topography (z scale 25 nm). (**b**) Δf (z scale 115 Hz). (**c**) KPFM (z scale 750 mV) high-magnification images of a layered P3OT lamellar structure (black square region in Figure 3). (**d**) Zone recognition by applying the segmentation procedure to the topography images. (**e**–**g**) Height, Δf and *SP* histograms of the different regions in (**d**) obtained from (**a**–**c**).

The higher lamellar structures (Figure 4) have flat layers of approximately constant height and up to five layers (in all the studied structures in HOPG). In order to study all the data measured by SFM—topography, frequency shift Δf and surface potential (KPFM data)—in more detail, we use the m2py library [50], which essentially allows us to detect “different regions” using a segmentation procedure explained in more detail elsewhere [51]. When this segmentation procedure is applied to the topography image, it clearly recognizes the different layers of the lamellar structure, as shown in Figure 4d, where each color codes the different regions recognized by the algorithm (including HOPG and the inter-lamellar structure), and in particular the different layers of the lamellar structure. We note that the colors of all histograms in Figure 4d are associated with data “recognized” by the segmentation procedure and correspond to some characteristic (topographic) feature and height value. As is easily recognized in the histogram of the topography data points measured (Figure 4e), we find that each layer has a height of around $d_{0}$ = 3.9 ± 0.3 nm. Comparing with X-ray structural studies [16,41] and previous SFM studies [19,46], each layer consists of two parallel P3OT chains, with the alkyl side chains perpendicular to the substrate, as shown schematically in Figure 1.

The frequency shift Δf image (Figure 4b) is mostly homogeneous within the lamellar structure, independently of the number of layers. This is confirmed in the corresponding histogram (Figure 4f), where all the layers of the lamellar structures show a similar Δf distribution, while the HOPG substrate and the random structure are significantly different. This indicates that all the layers are similar from a chemical nature point of view.

The KPFM data in the images and histograms show quite a complex structure, which is, interestingly, quite different from the frequency shift Δf image. This is surprising, since both frequency shift Δf and surface potential (KPFM data) should be related to the chemical composition. However, the lamellar structures, while appearing homogeneous in the Δf images, show a complex variability of *SP* even though the whole structure seems to be composed of the same P3OT chains with the same orientation. For the KPFM data in the images and histograms, we essentially find three main values: $SP_{0}=0$ for HOPG and the thin inter-lamellar structure, and two values in the the lamellar structures, $SP_{1}=-280$ mV and $SP_{2}=-500$ mV. The $SP_{1}$ region is correlated with the first layer in direct contact with the substrate, while $SP_{2}$ corresponds to the second and higher layers.

**Figure 5:**
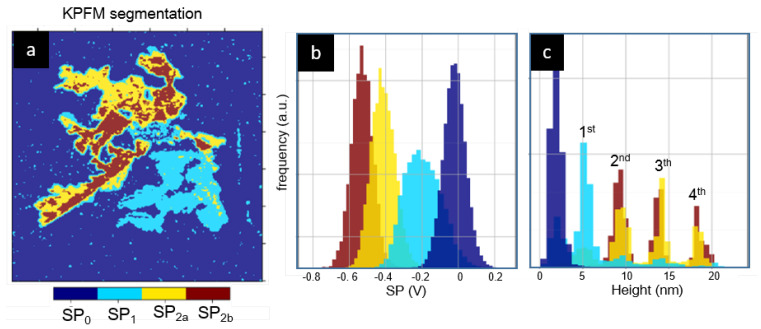
(**a**) Different *SP* regions identified under KPFM image segmentation. (**b**) *SP* histograms and (**c**) height histogram of the different *SP* regions identified in (**a**).

To obtain further information about the correlation between layer height and *SP* and its distribution within the lamellar structures, the segmentation procedure is applied to the KPFM images (Figure 5a). This analysis reveals that the $SP_{2}$ peak corresponding to the *SP* of higher layers is the superposition of two peaks that we named $SP_{2a}=-440$ mV and $SP_{2b}=-550$ mV (Figure 5b). In fact, we have found that, in all the layered structures, the second or upper layers are always composed of $SP_{2a}$ and/or $SP_{2b}$ type regions. Thus, regions with different *SP* may belong to the same layer and, conversely, regions with the same *SP* may correspond to different numbers of layers. Furthermore, the proportion of these two types of regions is very similar for all the layered structures, as shown in Figure 5c.

We note that, in addition to the random structure on the HOPG described above (represented in dark blue in Figure 4g), we have identified another region also as a random one (represented in brown in Figure 4g) that slightly covers some parts of the layers. This is because they have similar Δf and *SP* values. However, the prevalence of these “random on top” polymer structures is very low and we do not focus on it.

**Figure 6:**
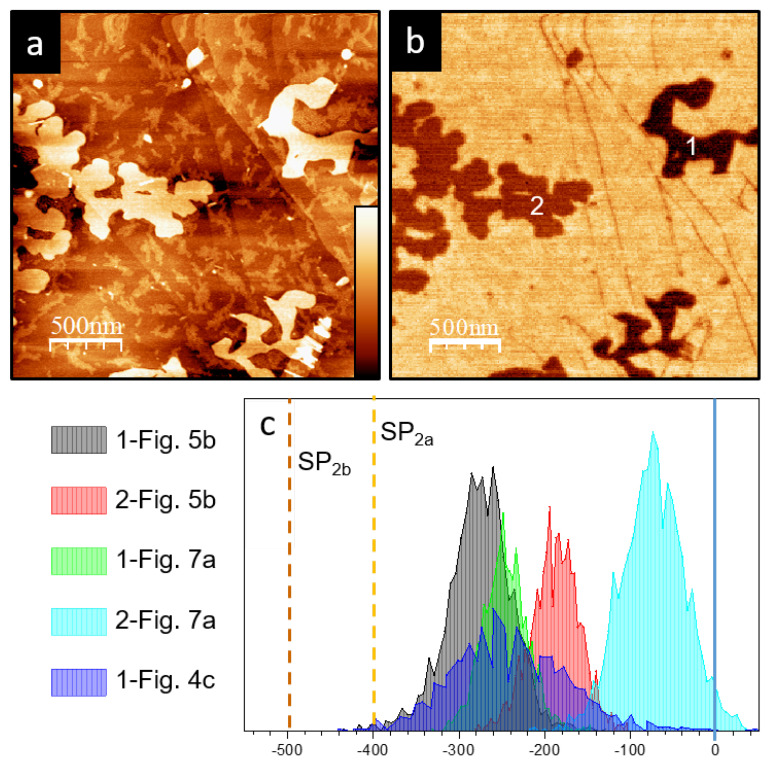
(**a**) Topography (z scale 25 nm). (**b**) KPFM (z scale ) images of a sample region where two one-layer P3OT lamellae with different *SP* values coexist. (**c**) *SP* distributions of the all one-layer P3OT structures shown in this work. Each structure has been identified in the corresponding KPFM image with a number. $SP_{2a}$ and $SP_{2b}$ mean values have been included to highlight that the one-layer *SP* is always higher.

**Figure 7:**
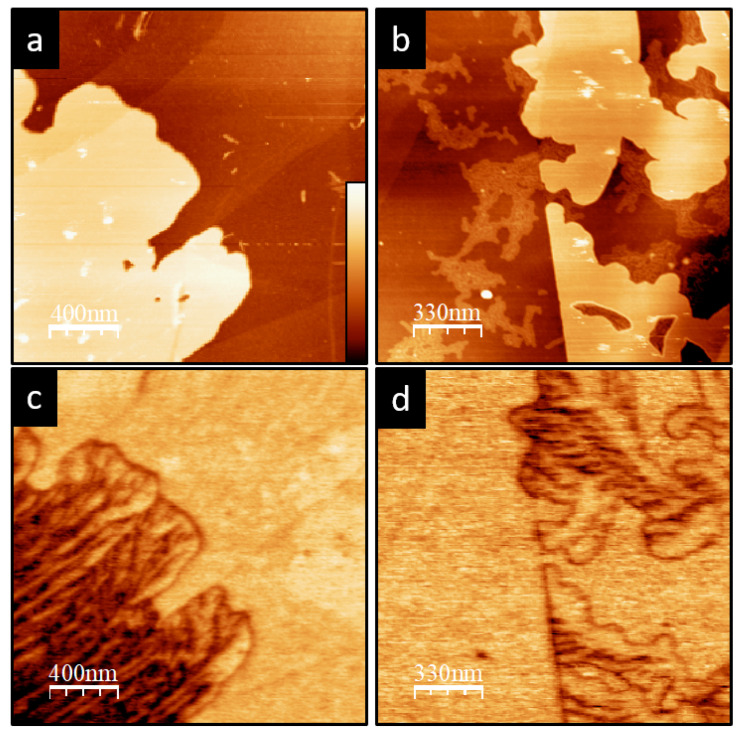
(**a**,**b**) Topography (z scale 25 nm). (**c**,**d**) KPM images (z scale 750 mV) of P3OT one-layer regions where the stripe-like substructures in the corresponding KPFM images are clearly seen.

While Figure 5c suggests that the first layer is uniquely correlated to the $SP_{1}$ value (around −280 mV), the inspection of different lamellar structures on the same sample, as well as our analysis of many other similar samples, shows that the first layer may present a large variety of *SP* values. This is particularly apparent in Figure 6a,b, where single-layer lamellar structures with different SPs are clearly distinguished. For the samples, we have found that the mean *SP* value associated with the first layer may vary from roughly 0 to −300 mV with respect to the HOPG. These values are summarized in Figure 6c, noting that they are always higher than $SP_{1}$ and $SP_{2}$ found in two or more stacked layers. In some cases, this rich variety of *SP* values may also appear over the same layer. However, in the first layer, instead of defined domains, a stripe-like substructure is resolved in the KPFM images (Figure 7c,d).

**Figure 8:**
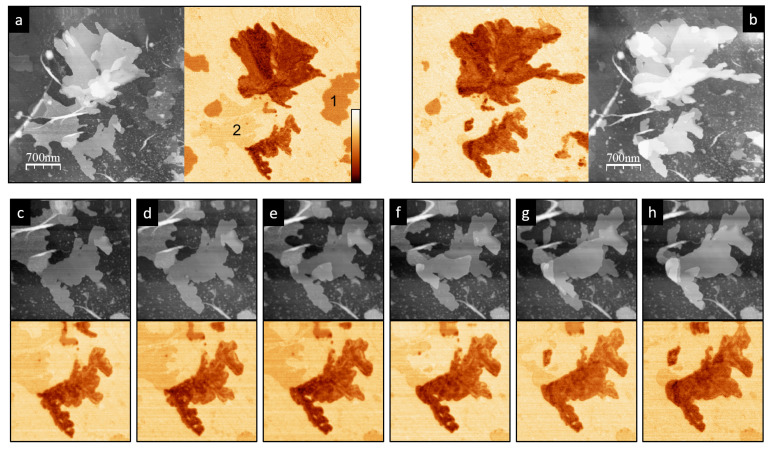
(**a**) Initial and (**b**) final topography (grayscale 35 nm) and KPFM images (color 750 mV) of a P3OT lamellar structure acquired in a time interval of 8 h. (**c**–**h**) High-magnification topography (upper panel) and KPFM (lower panel) images acquired at Δt=86 min.

Finally, we should note that remarkable dynamics have been observed on the P3OT structures, indicating high molecular motion under the acquisition conditions. This evolution is especially marked in the layered regions as compared with the disordered inter-layered structures. In these disordered regions, the face-on polymer backbone orientation probably favors the π−π backbone–HOPG interaction, limiting the molecule mobility. It was not the aim of this work to study the the detailed kinetics of the polymer chains, which may be induced by local surface heating due to the red laser of the beam deflection detector system. However, it is interesting to highlight that, as seen in Figure 8, in lamellar aggregates, the number of layers increases at the expense of the one-layer regions, which tend to disappear. Once again, this finding supports the notion that the first layer of the lamellar regions is clearly different, although, as shown above, the Δf signal does not reveal any different chemical nature.

## 4. Discussion and Conclusions

**Figure 9:**
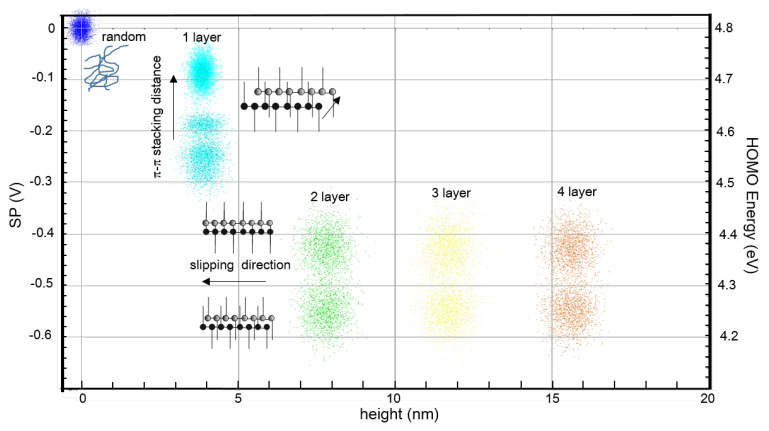
*SP* vs. height representation including all data shown in this work, together with the schematic representation of the proposed polymer aggregate type for each *SP* value. HOMO energy level has been calculated using the HOPG work function as the standard calibration reference in KPFM.

The previous results show that all the lamellar regions consist of one or several stacked layers, each with a height of approximately 4 nm. From a morphological point of view, the layers are similar, without significant variations in height, roughness or shape. On the contrary, the KPFM characterization reveals a rich and complex variety of *SP* values, which we summarize in Figure 9.

We have found that the *SP* of the first layer, in direct contact with the substrate, is clearly different from an electronic point of view as compared to the upper stacked layers. The *SP* value of this first layer is always larger than the *SP* of the on top layers, and closer to the value found in the loosely packed disordered regions. This suggests that the interchain spacing in the π−π c-axis registration direction may be larger in the first layer than in successive layers. This is in good agreement with the model developed by Spano et al. [35], which predicts that a reduction in the π−π stacking distance raises the HOMO level, decreasing the work function and, under our sign convention, decreasing the *SP*. Furthermore, the *SP* is not unique but it may vary by up to several hundreds of mV, not only between different layers but even within the same layer, indicating differences in molecular aggregation and supported by previous results that state that different π−π stacking distances might be possible in P3OT without changing the apparent layer thickness [52,53]. Moreover, the higher mobility of the first-layer polymer chains also supports a lower chain–chain interaction due to a lower packing density.

In the second or upper stacked layers, we always found two types of *SP* regions with characteristic values. These *SP* regions coexist within the same layer, independently of the number of layers. On the one hand, this indicates that layers that are not in direct contact with the substrate present similar electrostatic behavior. On the other hand, the fact that only two well-defined *SP* values are found with similar prevalence points towards the existence of two mostly similar, energetically favorable types of molecular aggregates. As their *SP* contrast is darker compared with that of the first layer, they have a lower energy HOMO level, probably due to a stronger intermolecular interaction and higher-density molecular packing. Assuming a roughly constant π−π stacking distance, the existence of different *SP* domains could be due to the slipping of successive chains along the c-direction. A side by side (head to tail) configuration enhances (reduces) π−π bond overlapping, lowering (raising) the HOMO level [9].

In conclusion, we have shown that semiconducting polymers and, in particular, P3OT crystalline lamellar structures might present a rich variety of *SP* values correlated with the fundamental state energies due to differences in molecular aggregation. We note that the size of the *SP* regions may vary from a few tens up to several hundreds of nanometers and coexist within single lamellar structures. These results show that, even at fixed preparation parameters, a polymer aggregation substructure emerges at the nanoscale. The contributions of these inhomogeneities to the optical signatures and the transport properties are still unknown, and this highlights the importance the complete characterization of the “dark” ground state.

## Data Availability

Not applicable.

## Supplementary Materials

The following supporting information can be downloaded at: https://www.mdpi.com/article/10.3390/ma15031212/s1, Supplementary material: Figure S1: Characterization of the substrate surfaces, Figure S2: Characterization of P3OT structures on ITO, Table S1: *SP* values obtained in P3OT lamellar structures on ITO.
