# Supplementary material for: Kelvin Probe Microscopy Investigation of Poly-Octylthiophene Aggregates
Source: Materials (Basel). 2022 Feb 6;15(3):1212. doi: 10.3390/ma15031212 (PMC8838950; doi:10.3390/ma15031212)
Supplement: Supplementary file 1 [file materials-15-01212-s001.zip › materials-1492694-supplementary.pdf]

Supplementary Material

Kelvin probe microscopy investigation of poly-octylthiophene aggregates

Joaquin Bermejo[1,†] , Jaime Colchero[1] and Elisa Palacios-Lidon [1]*

[1] CIOyN, Departamento de Física, Universidad de Murcia, 30100 Spain.
[†] Current address: Laboratoire de Physique de l'Ecole Normale Supérieure, ENS, Université PSL, CNRS, Sorbonne Université, Université de Paris, Paris, France.

*Correspondence: elisapl@um.es

1. HOPG and ITO characterization.

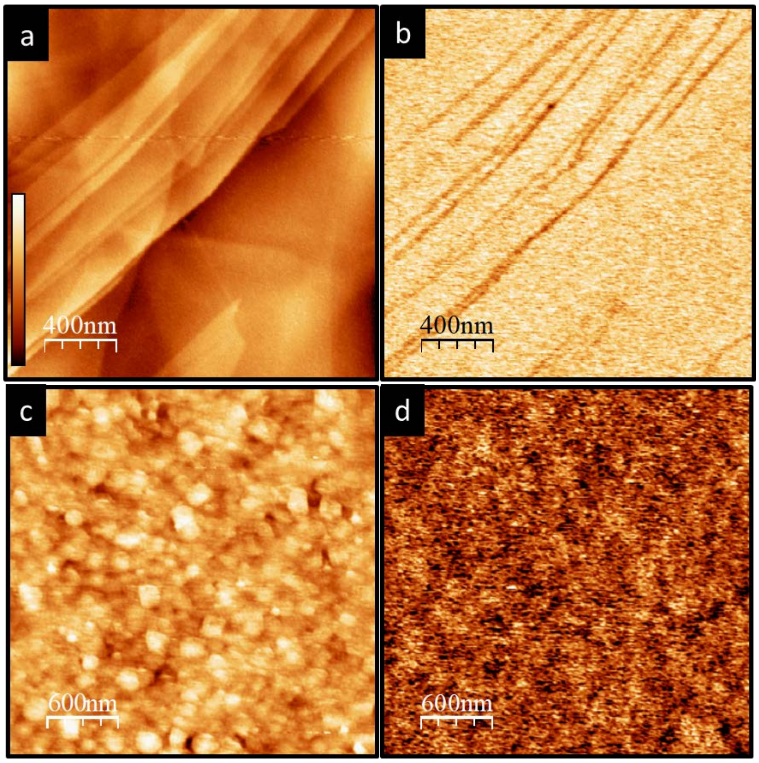

Figure S1. (a) Topography (z=3 nm) and **(b)** KPFM (z= 100 mV) images of a HOPG surface and **(c)** Topography (z=20 nm) and **(d)** KPFM (z= 100 mV) images of an ITO surface after deposition of a drop of pure toluene.

2. P3OT on ITO

Indium-tin-oxide is a highly degenerate n-type semiconductor, with a broad band gap, around 4 eV. Thus, it presents high transmission in the visible part of the electromagnetic spectrum and low electrical resistivity \cite{17}. These exclusive transparent and conducting properties have made of ITO an indispensable material in optoelectronics. P3OT low coverage samples has been prepared on flat ITO substrate.

Figure S2 shows that lamellar structures as well as polymer disordered inter-lamellar regions are also present in ITO, which gives an account of the tendency to self-assembly of P3OT regardless of the substrate. Due to the roughness of the ITO surface, the disordered polymer regions are not discerned on the topography image but they are clearly identified on the corresponding Δf image. Even more, it is possible to discriminate between ITO and the disordered structures in KPFM images what was not possible on HOPG.

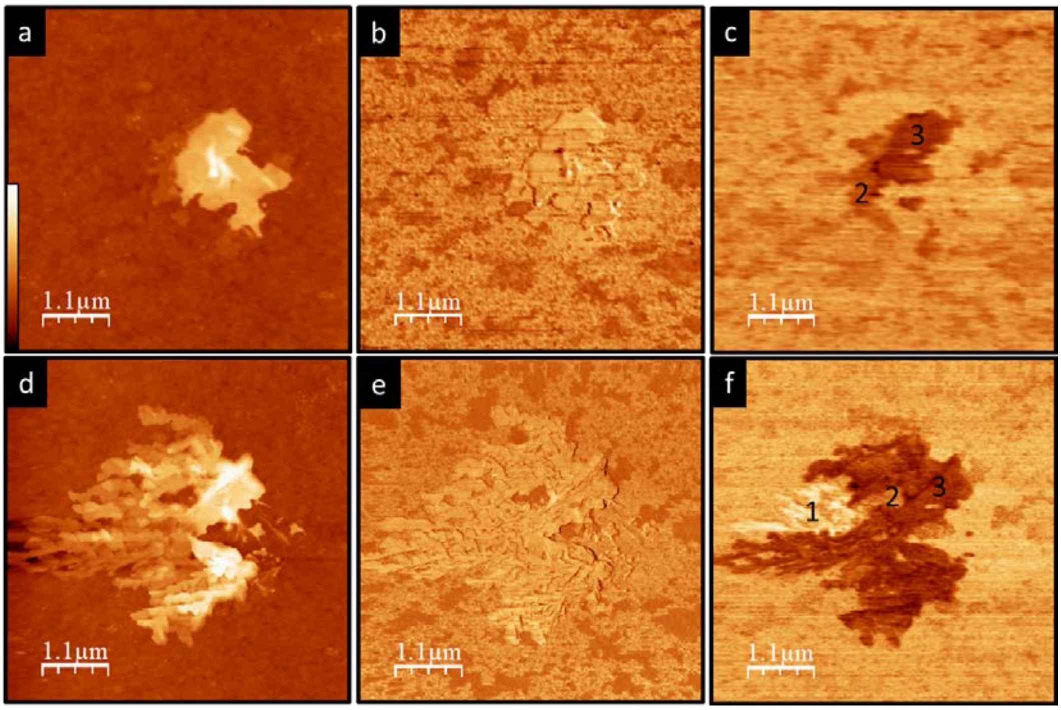

Figure S2. (a) and (d) Topography, (b) and (e) Δf and (c) and (f) KPFM images of two P3OT on ITO sample regions. Z scale is 50 nm, 174 Hz and 700 mV for topography, Δf and KPFM images respectively.

The lamellar structures consists, once again, on a superposition of layers of height around 4 nm, although on ITO, the number of stacked layers seem to be higher (up to 10 layers) than on HOPG. KPFM images yield a similar behavior for lamellar

structures than in HOPG: two SP domains also appear in in the second or upper stacked layers, while the first layer is different as shown in **Figure S2 (c) and (f)**.

This lead us to think that if the SP values are characteristic of the material, by choosing the same SP reference, we must obtain similar values regardless of the substrate. To do so, we use as reference the SP value of the disordered structure. SP values of regions identified in **Figure S2 (c) and (f)** are listed in **Table S1**

Region	SP (mV)
1	420 ± 70
2	-400 ± 50
3	-500 ± 60

Table S1. SP values referred to the random polymer structure of the regions marked in **Figure S2 (c) and (f)**

These values lead us to identify regions 2 and 3 of **Figure S2 (c) and (f)** with domains SP_{2a} and SP_{2b} found on HOPG. On the contrary, region 1 SP value has not been found before confirming that the first layer in direct contact with the substrate is different and may present different values probably due to substrate induced effects.
